# Major complications of post-chemotherapy retroperitoneal lymph node dissection in a contemporary cohort of patients with testicular cancer and a review of the literature

**DOI:** 10.1186/s12957-020-02032-1

**Published:** 2020-09-24

**Authors:** Christian Guido Ruf, Simon Krampe, Cord Matthies, Petra Anheuser, Tim Nestler, Jörg Simon, Hendrik Isbarn, Klaus Peter Dieckmann

**Affiliations:** 1 Department of Urology, Bundeswehrkrankenhaus Hamburg, Lesserstraße 180, 22049 Hamburg, Germany; 2 Department of Urology, Bundeswehrkrankenhaus Ulm, Oberer Eselsberg 40, 89081 Ulm, Germany; 3 Department of Urology, Albertinen Krankenhaus Hamburg, Suentelstrasse 11a, 22457 Hamburg, Germany; 4 Department of Urology, Bundeswehrzentralkrankenhaus Koblenz, Rübenacher Str. 170, 56072 Koblenz, Germany; 5 Department of Urology, Ortenau-Klinikum, Ebertplatz 12, 77654 Offenburg, Germany; 6 Martini Klinik, Universitätsklinikum Eppendorf, Martinistrasse 52, 20246 Hamburg, Germany; 7 Department of Urology, Asklepios Klinik Altona, Paul Ehrlich Strasse 1, 22763 Hamburg, Germany

**Keywords:** Testicular germ cell tumour, Retroperitoneal lymph node dissection, Lymphocele, Nonseminoma, Surgical complication

## Abstract

**Background:**

Post-chemotherapy retroperitoneal lymph node dissection (pc-RPLND) is one cornerstone in the clinical management of patients with nonseminomatous testicular germ cell tumours (GCT). A wide range of complication rates in this type of surgery is reported so far. We retrospectively evaluated the frequency of major complications by using the Clavien-Dindo classification and analysed the influence of various clinical factors on complication rates in pc-RPLND.

**Methods:**

We retrospectively analysed 146 GCT patients undergoing pc-RPLND. Complications of grade III–V according to the Clavien-Dindo classification occurring within 30 days after surgery were registered along with the following clinical factors: age, body mass index (BMI), duration of surgery, number of anatomic fields resected, side of primary tumour, histology of surgical specimen, histology of primary tumour, and total dose of cisplatin applied prior to surgery. For comparison, we also evaluated 35 chemotherapy-naïve patients with primary RPLND and 19 with laparoscopic RPLND. We analysed types and frequencies of the various complications as well as associations with clinical factors using descriptive statistical methods.

**Results:**

A total of 14.4% grade III–IV complications were observed in pc-RPLND, and 8.6% and 5.3% in primary and in laparoscopic RPLND, respectively. There was no perioperative mortality. Lymphocele was the most frequent adverse event (16% of grade III–IV complications). Operation time > 270 min (*p* = 0.001) and vital cancer in the resected specimen (*p* = 0.02) were significantly associated with higher complication rates. Left-sided resection fields involved two-fold higher complication rates, barely missing statistical significance (*p* = 0.06).

**Conclusions:**

Pc-RPLND involves a grade III–V complication rate of 14.4%. Prolonged operation time and vital cancer in the residual mass are significantly associated with higher complication rates. The Clavien-Dindo classification system may allow inter-observer variation in rating complication grades, which may represent one reason for the wide range of reported RPLND complication rates. RPLND represents major surgery and surgeons active in this field must be competent to manage adverse events.

## Introduction

Testicular germ cell tumours (GCT) represent the prototype of curable malignancies [1, 2]. The mainstays of therapy are cisplatin-based chemotherapy and retroperitoneal lymph node dissection (RPLND). The first ever reported RPLND was performed by the Swiss surgeon Theodor Kocher in 1883 [3]. Since this pioneering work, the procedure has received an ongoing number of surgical and perioperative refinements [4]. Today, RPLND is considered a safe surgical procedure in experienced hands [5].

There are several types of RPLND, the most frequent being post-chemotherapy RPLND (pc-RPLND) which is used for surgical resection of residual retroperitoneal masses after chemotherapy of nonseminomatous GCT [6, 7]. Primary RPLND (p-RPLND) is used less frequently in select cases with clinical stage (CS) 1 and with marker-negative CS 2, in which the operation is performed for diagnostic and therapeutic purposes [8–10]. In recent years, another type of RPLND has come into use, the laparoscopic RPLND (l-RPLND) [11].

Surgical complications are an ongoing issue in RPLND. With improvements of surgical and anaesthesiologic techniques the frequencies of complications have significantly decreased over the last decades. The reported rates of surgical complications of RPLND vary widely between 1% and almost 50% [12, 13].

The wide range of reported complication rates can be attributed to two aspects. First, although all types of RPLND are considered major abdominal surgery, pc-RPLND is technically much more challenging than primary resections due to desmoplastic reactions around the great abdominal vessels as an effect of chemotherapy [14, 15]. Sometimes these changes may hinder the access to the natural layers for dissection in the retroperitoneum, thus triggering the need for adjunctive surgical measures [16]. Therefore, pc-RPLND usually involves higher rates of complications than p-RPLND [17]. The second reason for the documented range of complications is methodological. Adverse events occurring during surgery or postoperatively may be rated quite differently by various surgeons. To objectify the appraisal of adverse events occurring postoperatively, the international Clavien-Dindo (CD) classification system of for assessing surgical complications was introduced in 2004 [18]. However, even when this gold standard is applied, interobserver variability still occurs [19], particularly with respect to minor complications [20, 21]. Furthermore, minor complications not affecting the over-all success of surgery may be ill-documented in archival patient files. Thus, retrospective evaluations of complication rates may overlook or misclassify these minor events and thus result in under-reporting of such complications.

The aim of the present study is to retrospectively analyse the surgical complication rates observed in a contemporary cohort of patients undergoing RPLND in two testicular cancer centres in Germany. To obtain meaningful data comparable to other series, we focused on the most frequent type of this surgery (pc-RPLND), and we rated the complications according to the CD classification system [18]. Furthermore, to avoid misclassifying minor complications in a retrospective chart review, we restricted our analysis to complications grade III to V of the CD system, which represent major complications requiring invasive or otherwise extended therapeutic measures. We also analysed whether particular clinical features are associated with certain complications.

## Methods

The electronic patient archives of Bundeswehrkrankenhaus Hamburg, Germany and Albertinen-Krankenhaus Hamburg, Germany, were retrospectively searched for cases with testicular cancer undergoing RPLND from 2000 to 2017. Three types of surgery were originally included in this study: post-chemotherapy RPLND (pc-RPLND) in cases with residual retroperitoneal masses after chemotherapy, open primary RPLND in CS 1 and CS2 cases (p-RPLND), and laparoscopic primary RPLND (l-RPLND). Four experienced surgeons performed these operations. Post-chemotherapy resections were performed as unilateral template resections in the presence of small residual masses. Larger residual masses usually required bilateral resections. p-RPLND was performed using the Indiana nerve-sparing technique in modified templates [22, 23]. In CS2 patients, bilateral dissections were performed with nerve sparing whenever possible. Laparoscopic resections were confined to unilateral templates. All patients were managed according to contemporary guidelines [24, 25].

Complications were defined as any adverse event occurring intraoperatively or within 30 days after surgery according to the CD system [18]. Adjunctive procedures during surgery such as nephrectomy, bowel resection or vascular repairs were not considered as complications when they were required for completeness of excision. The following data were abstracted from the files: type of RPLND (p-RPLND, l-RPLND, pc-RPLND), histology of the primary testicular tumour (nonseminoma/seminoma/teratoma as component of nonseminomatous tumour), histology of resected specimen (vital GCT, teratoma, necrosis), patient’s age, body mass index (BMI), duration of the surgical procedure, number of topographic fields resected according to the Weissbach field classification [26], laterality (left/right side), and in pc-RPLND also cumulative dosage of cisplatin applied prior to surgery (< 500 mg/500–700 mg/> 700 mg); complications (yes/no), grade of complication according to the CD classification [18].

For final analysis, only major complications were included (grade III–V CD system [18]). In patients developing more than one complication, only the most severe event was included into the analysis. Our analysis involved four steps: (1) we listed the types of adverse events observed. (2) The frequency of grade III–V complications was assessed according to the three types of surgery. (3) In pc-RPLND, correlations between complications and clinical factors were analysed. Due to the retrospective evaluation, information regarding the various clinical factors was not available in all patients and thus, sample sizes of subgroups vary in this analysis due to missing data. (4) We compared our findings with complication rates of RPLND documented in the literature from 2000 to 2020.

The statistical analysis was computed with SPSS software (Version 20, IBM, USA), mainly employing descriptive statistical methods. The chi-squared test was used for statistical comparisons of proportions. A *p* value < 0.05 was considered significant.

Ethical consent was obtained from the institutional ethical committee (AEK, U03, Hamburg 12 June 2016). All study activities conformed to the Helsinki Declaration of the World Medical Association (as amended by the 64^th^ General Assembly, 2013).

## Results

**Fig. 1:**
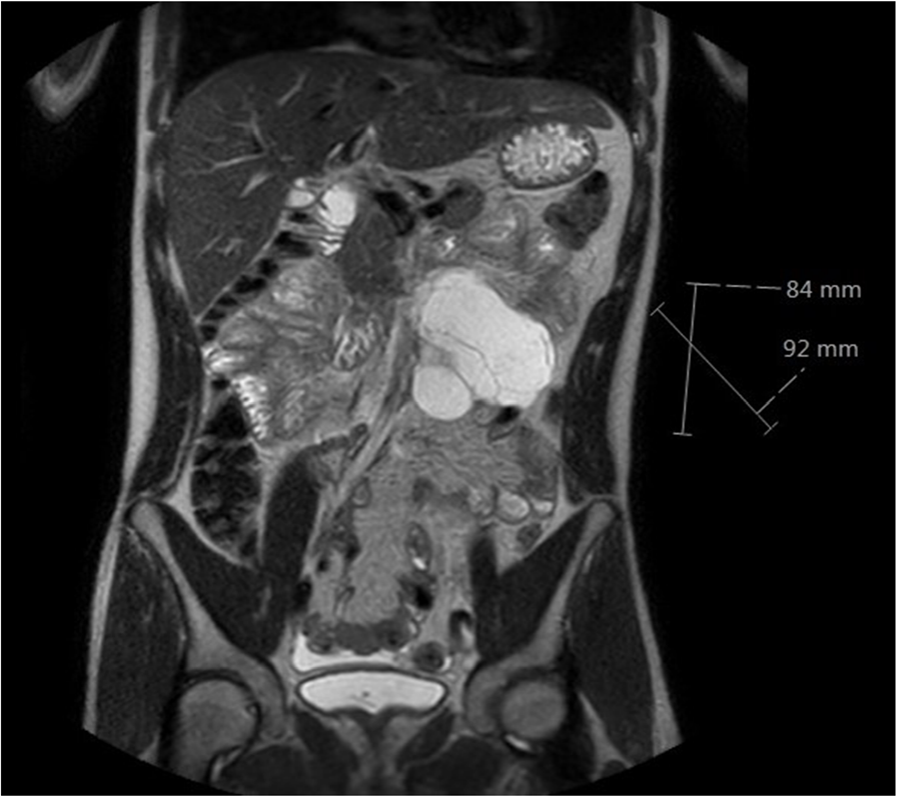
Left-sided multi-chambered intraabdominal lymphocele arising after post-chemotherapy RPLND. Magnetic resonance imaging, T2-weighted imaging, coronal sectioning. This lymphocele resolved spontaneously within 3 months

A total of 201 patients were identified, thereof 147 with pc-RPLND, 35 with p-RPLND and 19 with l-RPLND. Clinical details of these patients are listed in Table 1. One patient with pc-RPLND was excluded from further analysis due to missing data on complications. A total of 200 patients were eligible for further analysis. About one quarter of the patients had been included in previous reports [9, 27]. Histologic examination of surgical specimens of pc-RPLND yielded necrosis/fibrosis, teratoma, and vital cancer in 44.1%, 44.8%, and 11.1%, respectively. Among the entire cohort of 200 patients, we identified a total of 98 patients (49%) where any type of complication was documented, thereof 25 patients (12.5%) with grade III–IV complications (complications requiring surgical or interventional measures) according to the CD classification. There was no grade V complication (perioperative death). Symptomatic lymphocele (Fig. 1) was the most frequent grade III–IV adverse event observed in 4 patients (16% of all grade III–IV complications, 2% of all patients included in the cohort). A detailed synopsis of the clinical features of major complications is presented in Table 2.

**Table 1:** Clinical details of patients included in the study

	Post-chemotherapy RPLND (*n* = 147)	Primary RPLND (*n* = 35)	Laparoscopic RPLND (*n* = 19)
Age (years) median (IQR) range	30 (23–37) 14–82	29 (24–37) 16–57	31 (25–35) 17–55
BMI (kg/m^2^) median (IQR) range	25.2 (22.6–28.4) 17–37	25.8 (21.7–27.7) 17–46	26.0 (23.1–28.7) 20–38
Seminoma as primary tumour	17/147 (11.6%)	1/35 (2.9%)	0/19 (0%)
Teratoma predominant in primary tumour	18/147 (12.2%)	5/35 (14.3%)	4/19 (21.1%)
Operation time (min) median (IQR) range	269 (215–378) 66–735	246 (201–329) 50–440	165 (106–207) 57–234
Median number of fields resected ^a^	5	4	4
Primary tumour right-sided	68/147 (46.3%)	14/35 (40%)	7/19 (36.8%)

^a^According to Weissbach Field Classification [26]

**Table 2:** Synopsis of types of grade III–IV complications according to Clavien-Dindo classification [18] observed in all RPLNDs (*n* = 200) ranked by over-all frequency

Type of complication	(***n***) total number of events	(%) of all RPLNDs (***n*** = 200)	(***n***) in pc-RPLND patients	(%) in pc-RPLND patients (***n*** = 146)	(%) of all grade III–IV complications
Lymphocele	4	2%	3	2.1%	16%
Ileus	3	1.5%	3	2.1%	12%
Intraoperative trauma to ureter	3	1.5%	3	2.1%	12%
Wound healing problems	3	1.5%	2	1.4%	12%
Postoperative haemorrhage	2	1%	0	0%	8%
Pneumonia	1	0.5%	1	0.7%	4%
Gastroparesis	1	0,5%	1	0.7%	4%
Ectasia of renal pelvis	1	0.5%	1	0.7%	4%
Pancreatitis	1	0.5%	1	0.7%	4%
Wound opening with bowel protrusion	1	0.5%	1	0.7%	4%
Retroperitoneal haematoma	1	0.5%	1	0.7%	4%
Pneumothorax	1	0.5%	1	0.7%	4%
Cardiac arrhythmia	1	0.5%	1	0.7%	4%
Atelectasis of lower lung lobe	1	0.5%	1	0.7%	4%
Systemic inflammatory response syndrome (SIRS) with disseminated intravascular coagulation (DIC)	1	0.5%	1	0.7%	4%

A comparison of the complication rates between the three types of RPLND revealed a higher rate of grade III–V complications in pc-RPLND (14.4%), compared to primary (8.6%) and laparoscopic resections (5.3%). However, this difference is not statistically significant (Table 3).

**Table 3:** Major complication rates (grade III–V, Clavien-Dindo classification) in relation to type of RPLND

	*n*	Complication grade III–IV *n* (%)	*p* value *
Post-chemotherapy RPLND	146	21 (14.4%)	
Primary RPLND	35	3 (8.6%)	0.39
Laparoscopic RPLND	19	1 (5.3%)	

* chi squared test

There was no significant change of grade III–IV complication rates over time. During the first and the second half of the time span of our survey (2000–2017), the complication rates were 13.2% and 15.1%, respectively.

The correlations of complications in pc-RPLND and various clinical factors are listed in Table 4. Significantly higher complication rates were observed in patients with duration of surgery exceeding 4 ½ h and in those with viable cancer in the resected specimen. A strong trend was found for a higher complication rate in left-sided compared to right-sided resections (*p* = 0.06). A slightly weaker trend towards increased complication rates was observed in obese patients with BMI ≥ 30 kg/m^2^ (*p* = 0.18). None of the other clinical factors evaluated were associated with complication rates.

**Table 4:** Grade III–IV complications of pc-RPLND according to Clavien-Dindo classification in relation to clinical factors

Clinical factor evaluated	Eligible total (*n*)	Categories (eligible in category, *n*)	Patients with complications (*n*), (%)	*p* value^b^
Age	143	≤ 30 years (*n* = 76)	10 (13.2%)	0.59
		> 30 years (*n* = 67)	11 (16.4%	
BMI	131	≤ 29 kg/m^2^ (*n* = 103)	12 (11.7%)	0.18
		≥ 30 kg/m^2^ (*n* = 28)	6 (21.4%)	
Laterality of primary	137	Right-sided (*n* = 68)	6 (8.8%)	0.06
		Left-sided (*n* = 69)	14 (20.3%)	
Duration of surgery	67	≤ 269 min (*n* = 34)	(0%)	0.001
		≥ 270 min (*n* = 33)	9 (27.3%)	
Cisplatin dosage	93	< 500 mg (*n* = 18)	2 (11.1%)	0.83
		500–700 mg (*n* = 45)	4 (8.9%)	
		> 700mg (*n* = 30)	4 (13.3%)	
Number of resection fields ^a^	139	≤ 5 (*n* = 82)	10 (12.2%)	0.54
		> 5 (*n* = 57)	9 (15.8%)	
Teratoma in primary	92	Yes (*n* = 18)	2 (11.1%)	0.83
		No (*n* = 74)	7 (9.5%)	
Histology of resected specimen	145	Necrosis/fibrosis (*n* = 64)	7 (10.9%	0.02
		Teratoma (*n* = 65)	8 (12.3%)	
		Viable cancer (*n* = 16)	6 (37.5%)	

^a^Resection fields according to Weissbach field classification [26]

^b^Chi-squared test

## Discussion

This study confirms that pc-RPLND with a rate of 14.4% of grade III–V complications according to the CD classification [18] is justifiably considered major surgery. Factors influencing the complication rates are duration of surgery ≥ 4 ½ h, vital cancer in the resected specimen and, surprisingly, left-sided post-chemotherapy resections. The most frequent type of complication is the occurrence of lymphoceles.

A vast number of studies on various aspects of RPLND have been published since the beginning of this century. However, only few provided specific details on complication rates. An overview of contemporary studies providing data on grade III–V complications utilizable for comparison is given in Table 5. Five studies reported higher rates than the present series [27–31], 10 lower [8, 12, 15, 32–39].

**Table 5:** Complication rates of pc-RPLND according to grade III–V of the Clavien-Dindo classification—survey of the literature

						Complications	
					Caseload	Grade III–IV	Grade V
First author	Ref #	Year	Institution/country	n	pts/year	%	%
Hendry ^a^	32	2002	London /UK	442	19.2	5.6	1.1
Pottek^a^	27	2004	Hamburg/Ger	207	10.4	15.9	0
Spiess^a^	28	2006	Houston/USA	236	10.3	23.3	1.3
Williams^a^	8	2009	Boston/USA	92	11.5	9.8	0
Flechon^a^	29	2010	Lyon/France	151	13.0	14.6	0.7
Subramanian	15	2010	Cleveland/USA	96	13.0	7	1
Luz	30	2010	Montreal/Can	73	4.9	19.2	0
Djaladat	33	2012	Los Angeles/USA	85	12.1	1.2	0
Cary	12	2015	Indiana/USA	755	83.9	1.3	0.3
Considine	34	2016	Dublin/Ireland	78	n.a.	9.0	0
Dusaud	35	2016	Multicenter/France	469	n.a.	2.0	0.2
Wells	31	2016	Multicenter/UK	162	n.a.	17.2	0
Paffenholz	36	2018	Cologne/Ger	162	10.8	7.4	0
Hiester	37	2019	Düsseldorf/Ger	171	10.7	5	1
Gerdtsson	38	2020	Multicenter Sweden/Norway	97	5.0	12.3	0.3
Blok	39	2020	Utrecht/NL/2 ctrs	124	n.a.	9.7	1.6
Present series		2020	Hamburg/Ger/2 ctrs	146	11.1	14.4	0

*n.a* not available, *ctrs* centers, *pts* patients, *Can* Canada, *Ger* Germany, *NL* Netherlands, *UK* United Kingdom

^a^These studies did not report according to the Clavien-Dindo system. In these series, data were rated in analogy to the system, and deaths were considered grade V

Caseloads were calculated from published reports according to numbers of patients and time span of patient accrual

No perioperative mortality was observed in our series, which is consistent with other recent reports [8, 13, 27, 30, 31, 33, 34, 36]. Low mortality rates of 0.20 to 1.6% [12, 29, 32, 35, 38, 39] were reported in some other studies (Table 5). In a Dutch series, one death due to anaesthesiological problems was reported [40]. Based on these data, it may be concluded that perioperative death is exceptionally rare in pc-RPLND.

The reported range of major complications is amazingly wide, from as low as 1.2% to up to 23.3% (Table 5). There are at least three factors that may explain these widely divergent rates: (1) surgical experience, (2) patient-related factors and extent of the surgical procedure, (3) methodological discrepancies with regard to adherence to the CD classification system.

(1) The data compiled in Table 5 indicate that surgical experience might be an important factor influencing the incidence of adverse events. In our opinion, the average number of cases operated per year (caseload) should be considered an indicator for experience, rather than the total number of patients involved in a report. Accordingly, the two studies with the highest caseloads (84/year and 19.2/year) reported the lowest incidences of complications [12, 32]. Conversely, the two studies reporting the highest complication rates had much lower caseloads of 4.9/year and 10.3/year [28, 30]. The significance of individual surgical experience is certainly paramount [41], and therefore, it has repeatedly been advocated that major surgery such as RPLND should be performed only in dedicated centres of excellence [5, 42–45]. In conclusion, one reason for the great variance among reported complication rates may be surgical experience.

(2) One patient-related factor that was shown to be associated with higher complication rates is the presence of vital cancer in the resected specimen. This association might be attributed to the size of resected tumours, because larger residual masses contain vital cancer more frequently than small masses [46]. As size of the residual mass is significantly associated with adjunctive surgical measures [47], tumour size could be the underlying reason for the association of vital cancer in the specimen with complication rates.

Operation time over 4 ½ hours was also shown to be associated with higher complication rates (Table 4). However, it is rational to assume that operation time itself is not the cause of complications; rather, prolonged operation time is caused by complex intraoperative situations that may trigger complications.

Generally, there is strong evidence for a significant association between age and comorbidity with the frequencies of surgical complications in major urological procedures [48]. However, testicular cancer is a malignancy typically afflicting young men with no or little comorbidity. Accordingly, in patients aged ≥ 31 years, we did not observe higher rates of grade III–IV complications. In contrast, other reports considered age as a risk factor for intraoperative complications. In a national cohort in the USA, a higher number of adjunctive surgical measures had been employed in patients 35 years or older undergoing RPLND [16]. However, intraoperative adjunctive measures do not directly translate into higher rates of postoperative complications as shown in the current study.

Obesity is another well-known factor impacting the incidence of adverse events upon surgery, mainly wound healing issues, thromboembolic events and respiratory postoperative problems [49, 50]. Accordingly, we found a two-fold higher rate of grade III-IV complications in patients with BMI ≥ 30 kg/m^2^, but the difference was not statistically significant, probably due to small sample size.

Curiously, in the present series, left-sided procedures involved a two-fold higher complication rate than right-sided RPLND, and to our knowledge, this observation has not been reported previously. The result closely misses statistical significance (*p* = 0.06). Yet, the association seems reasonable because left-sided template resections sometimes require extended mobilisation of small bowels and colon, and this surgical manoeuvre may precipitate complications, e.g. small bowel obstruction and postoperative ileus. In accordance with our finding, a Scandinavian study reported a higher rate of adjunctive measures in left-sided resections [38].

In summary, patient-related factors such as comorbidities, duration of surgery, laterality of procedure, and histology of resected specimen may affect complication rates. Varying incidences of patient-related factors in studies reported in the literature may therefore partly explain the wide range of complications in pc-RPLND.

(3) The third factor affecting the reported complication rates of RPLND is the methodology for the assessment of complications. For comparing surgical complication rates among institutions or studies it is paramount to define and classify adverse events in a uniform and objective manner [51]. Although strong efforts have been made to classify complications in an unambiguous manner by introducing the CD classification [18], there is still evidence for interobserver discrepancies regarding the assessment of adverse perioperative events [19, 20] particularly concerning surgical site infections [52]. This was demonstrated in a Canadian survey among urologic surgeons which found an interrater agreement rate of 75% [53].

The CD system is largely based on the extent of measures to manage complications [54]. Major complications necessitating clearly defined interventions usually involve high interobserver agreement rates. Conversely, minor complications of grade I–II requiring only conservative measures or just observation involve much more interobserver variation. This was demonstrated in an international survey of 98 urologists, who rated 70 different complications of nephrolithotomy [21]. In view of several weaknesses of the CD system, modifications have been suggested [55], but currently it is still considered the gold standard [54].

In our evaluation of complications of RPLND, we rated a high number of grade I–II complications. In final analysis, we realized that most of these events originally considered as complications were indeed asymptomatic lymphoceles that had been detected incidentally on routine postoperative ultrasound examinations. Other findings such as redness and anejaculation also did not alter the postoperative course. Clearly, these findings are prone to interobserver variation. Therefore, we elected to focus on clearly defined major complications (grade III–V of the CD classification). This is based on empirical evidence that interobserver agreement rates are highest regarding grade V complications (death) and decrease in lower complication grades [21].

With respect to the widely divergent complication rates of pc-RPLND (1.2–23.3%) summarized in Table 5, we must certainly acknowledge that apart from surgical expertise and patient-related factors, methodological problems of rating the various types of complications may also contribute to the large variance of reported results. Accordingly, it is not yet empirically possible to clearly define a benchmark or an acceptable upper limit of complication rates.

The most frequent complication in our series was lymphocele formation (16% of all grade III–IV complications in all types of RPLND). Accumulation of lymphatic fluid in the abdomen is thought to derive from dissecting and opening of lymphatic vessels without proper ligation during resection of retroperitoneal masses. Whether perioperative low-dose heparin administration for prophylaxis of thromboembolic events may contribute to the development of lymphoceles remains unclear. Vahlensieck was the first to document postoperative retroperitoneal lymphoceles following p-RPLND in 1973 [56]. Low-dose radiotherapy was used to manage the complication in those cases. In contemporary series of p-RPLND and pc-RPLND, reported rates of lymphocele formation range from 0 to 14.6%, with the majority of studies reporting low rates of 1.5 to 5% [15, 27, 40, 47, 57], but some also report higher rates of 10.9% [30], 11% [38], and 14.6% [29]. Probably, the wide range of lymphocele occurrences reported can be attributed to differences in coding systematics. One may assume that lymphoceles were not accounted for at all in many of the previous reports, unless invasive interventions such as insertion of drainage tubes were required. Accordingly, the case shown in Fig. 1 had only very mild abdominal discomfort according to grade I or II of the CD system despite the rather high volume of lymphatic fluid accumulating in the abdominal cavity. This lymphocele resolved completely within 3 months without specific measures.

The main limitation of our study is the retrospective mode of data acquisition. As not all of the adverse events were unequivocally documented in the patient files, some events might have been missed or graded inappropriately. Due to small numbers of events, statistical analysis did not reach significant results in some of the calculations.

## Conclusions

Complications in pc-RPLND represent a multifaceted problem because adverse events may result from a large number of factors. In the present study, the rate of grade III–IV complications according to the CD system was 14.4% (no postoperative mortality), which is in line with most of the previous reports on this subject. We conclude that it is problematic to define a benchmark or an upper (acceptable) limit of complications because at least three factors may affect complication rates: first, patient-related factors including extent of surgery and comorbidities, second, methodological considerations in rating clinical events as complication, and third, surgical expertise. All in all, retroperitoneal lymph node dissection in patients with testicular cancer represents major surgery with a high potential of adverse events. Any surgeon active in this field must be prepared to manage adverse events occurring intraoperatively or during the postoperative course. Patients with testis cancer requiring such type of surgery are probably best advised to attend high-volume specialised referral centres.

## Data Availability

The datasets used and/or analysed during the current study are available from the corresponding author on reasonable request.

## Abbreviations

RPLND: Retroperitoneal lymph node dissection
p-RPLND: Primary (open) RPLND
l-RPLND: Laparoscopic RPLND
pc-RPLND: Post-chemotherapy RPLND
GCT: Germ cell tumour
BMI: Body mass index
CD: Clavien-Dindo
